# Smartphone-Based Food Diagnostic Technologies: A Review

**DOI:** 10.3390/s17061453

**Published:** 2017-06-20

**Authors:** Giovanni Rateni, Paolo Dario, Filippo Cavallo

**Affiliations:** The BioRobotics Institute, Scuola Superiore Sant’Anna, Viale Rinaldo Piaggio 34, 56025 Pontedera (PI), Italy; paolo.dario@santannapisa.it (P.D.); filippo.cavallo@santannapisa.it (F.C.)

**Keywords:** mobile diagnostics, food analysis, smartphone sensor, lab-on-smartphone, food safety, on-site detection, biosensors, spectroscopy, IoT, cloud computing

## Abstract

A new generation of mobile sensing approaches offers significant advantages over traditional platforms in terms of test speed, control, low cost, ease-of-operation, and data management, and requires minimal equipment and user involvement. The marriage of novel sensing technologies with cellphones enables the development of powerful lab-on-smartphone platforms for many important applications including medical diagnosis, environmental monitoring, and food safety analysis. This paper reviews the recent advancements and developments in the field of smartphone-based food diagnostic technologies, with an emphasis on custom modules to enhance smartphone sensing capabilities. These devices typically comprise multiple components such as detectors, sample processors, disposable chips, batteries and software, which are integrated with a commercial smartphone. One of the most important aspects of developing these systems is the integration of these components onto a compact and lightweight platform that requires minimal power. To date, researchers have demonstrated several promising approaches employing various sensing techniques and device configurations. We aim to provide a systematic classification according to the detection strategy, providing a critical discussion of strengths and weaknesses. We have also extended the analysis to the food scanning devices that are increasingly populating the Internet of Things (IoT) market, demonstrating how this field is indeed promising, as the research outputs are quickly capitalized on new start-up companies.

## 1. Introduction 

Mobile diagnostics is gaining more and more attention in healthcare, environmental monitoring, and agro-food sectors, allowing rapid and on-site analysis for preliminary and meaningful information extraction. The aim is to bypass the use of expensive and bulky instrumentation-based routine tests, performed by trained personnel, with the goal of cost saving and time efficiency. Not by chance, recently a Horizon Prize regarding the challenge of developing a rapid non-invasive food scanning device has been launched [1], reflecting the fact that it comes to a hot topic not only in research, but also for the market. Moreover, this approach could have a substantial positive effect on health, environmental, and food diagnostics technologies in both developed and developing countries, leading to a *democratization* in measurement science, thanks to the massive volume of mobile phone users spread globally [2]. Indeed, the ubiquitous availability of smartphones throughout the world enables a broad accessibility. Smartphones can be deployed in a variety of environments, including remote or underdeveloped rural regions. For example, the current methods for ensuring food safety rely on routine, but highly resource-intensive laboratory-based examination of chemicals and/or foodborne pathogens. In remote areas, where resources are scarce, sending specimens to an analysis laboratory can be difficult. Smartphone-based analytical platform, instead, could bypass these logistic issues via on-site testing or remote confirmation of detection [3].

Smartphones are equipped with numerous components that can be employed for measurement and detection, such as a fast multicore processor, digital camera, battery, visual display, and intuitive user interface. Smartphones also possess several wireless data transfer modalities (e.g., cellular data service, Wi-Fi, Bluetooth), allowing test results to be displayed immediately to the user and/or transmitted to cloud databases. Nevertheless, smartphones can-not function alone as laboratory instruments. Rather, they need to be augmented by other accessories. Such augmented devices have great potential as mobile diagnostic platforms for food analysis. In recent years, many external sensor modules have been designed and integrated with smartphones to extend their capabilities for extracting more-sophisticated diagnostic information. These portable, low-cost devices have the potential to run routine tests, which are currently performed by trained personnel using laboratory instrumentation, rapidly and on-site, thanks to the global widespread use of cellphones. In the field of food, this ability translates to improved awareness of what we eat. Examples include empowering the mobile phone of an allergic subject with personalized diagnostic capability, or allowing rapid inspection in the case of suspected contamination by foodborne pathogens or other hazardous contaminants. Moreover, even the agro-food sector could benefit from the development of portable lab-on-smartphone platforms, allowing on-field extraction of valuable data about a crop’s maturity and health state. With advances in micro-manufacture, sensor technology, and miniaturized electronics, diagnostic devices on smartphones will be used increasingly to perform biochemical detections in healthcare diagnosis, environmental monitoring, and food evaluation in the near future [4].

Much effort has been directed toward using the mobile phone as a sensing device, as described in Li et al. [5]. The increasing number of measurement applications on smartphones is due to their growing capabilities of sensing physical quantities by leveraging new embedded sensors or through wireless and wired connection possibilities and smart visual interfaces, to receive measurements from different external systems [6]. While many works have focused on the development of point-of-care systems for biomedical purposes as reported in Xu et al. [7], smartphone diagnostic platforms also have emerged in other important applications. Recent existing reviews by Roda et al. [8], Liu et al. [9], and Yang et al. [10] have described these advancements. However, to the best of our knowledge, a review focused on the specific application of smartphone-based diagnostic platforms for food analysis is not yet available.

To fill this gap, we provide a review of analytical devices able to enhance the ordinary smartphone with diagnostic capabilities. First, we explain the review methodology we pursued to create the provided bibliography. Then, we provide the reader with an overview of the latest relevant research works categorized according to the diagnostic method, with each working principle explained. We also mention the recent commercial exploitations in this sector, and provide the most interesting solutions. We conclude by discussing our perspectives on the current limitations, challenges, and future directions of this novel, emerging field of research.

## 2. Materials and Methods 

This systematic review collects the latest developments in smartphone-based food analytical systems and food bio-sensing approaches reported from 2002 to the present. The bibliography has been built upon searches in the Web of Science, US National Library of Medicine (PubMed), Scopus, and ScienceDirect databases. Reference lists of included articles and relevant review articles were examined to identify every study which the electronic search strategy may have missed. The search queries included the following terms: “(smartphone OR cellphone) AND (portable OR mobile OR instrument OR sensor OR device OR platform) AND (sensing OR testing OR analysis OR detection OR measurement OR monitoring) AND (food OR fruit OR meal OR beverage)”.

Application of these search keys provided 115 hits in the Web of Science in the field ‘Topic’, 126 hits in PubMed in ‘All fields’, 168 hits in Scopus in ‘Article title, abstract, keywords’ fields, and 31 hits in ScienceDirect in ‘Article title, abstract, keywords’ fields.

Articles obtained from the database search were exported using Mendeley reference manager. After deletion of overlaps, this process resulted in a total of 292 records that met the search criteria.

Titles and abstracts retrieved by the electronic search were read first, to identify articles deserving a full review. Papers not written in English were excluded. Improvement of works presented by same authors were counted as one. After application of a title filtering process, 223 works were excluded because they were out of topic; 69 works remained at this stage. 

**Figure 1:**
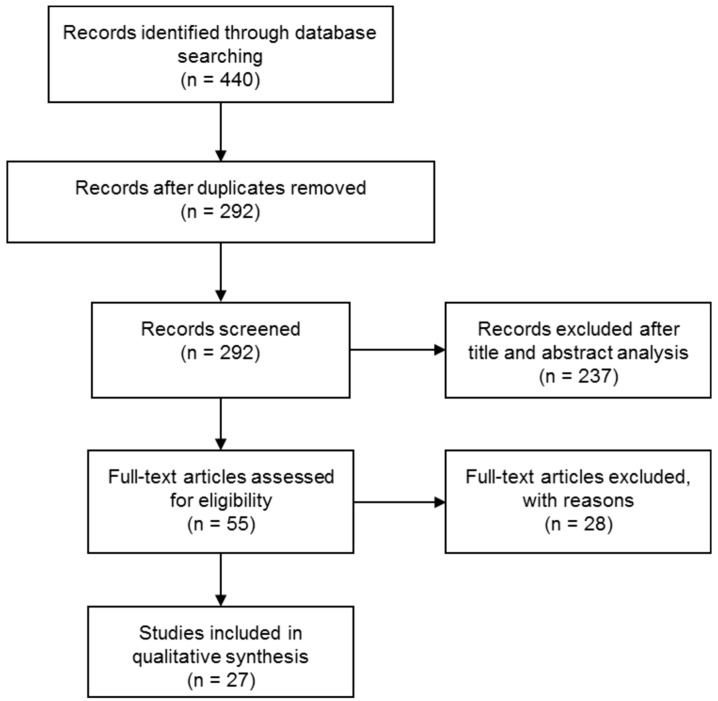
Flow diagram of the paper selection process.

After abstract analysis, nine works were discarded because they were out of topic, and five were found to be interesting review papers, used to contextualize the descriptions. Then, a full-text assessment was carried out, resulting in 23 studies based on smartphone use for food intake tracking and dietary/diabetes management. These were excluded, because the focus of this review were works describing the development of sensing modules used in combination with smartphone to perform diagnostic analysis. Thus, the final list included 27 published studies, which satisfy the selection criteria. The year of publication spans from 2012 to the present. This review was performed in February and March 2017. The studies selection process is shown in Figure 1.

## 3. Results

Smartphone-based food diagnostic approaches can be divided into two categories: lab-on-smartphone biosensors and smartphone optical and spectroscopy. Studies are presented in chronological order for each detection method. An explanation is provided of the technique employed and the added value the integration of a smartphone in the loop provides to the approach.

### 3.1. Lab-On-Smartphone Biosensors

Biosensors are analytical devices that integrate a bio-receptors with an appropriate transducing method to detect analytes of interest. The specific interaction between the target analyte and the receptor produces an output measurable signal, which highlights the presence of the sought element. Thus, this approach has high selectivity, since direct detection of the target analyte is achieved. However it always includes an invasive sample pre-treatment phase to give rise to transduction. Biosensors allow low-cost, and fast analysis, with results in a few minutes, and show perspectives for miniaturization and portability. Taking advantage of the combined use of smartphone and adapted biochemical assay, biosensor-based analytical systems are promising tools for on-site detection of analytes including contaminants, drugs, pesticide residues, and foodborne pathogens. A detailed overview of the revised Lab-On-Smartphone Biosensors works is provided with Table 1.

#### 3.1.1. Fluorescence Imaging Using Smartphone

Fluorescence imaging is the visualization of fluorescent dyes as labels for biological or chemical molecules of interest. It enables a wide range of experimental observations including the location of gene expression, protein expression and molecular interactions in cells and tissues. To label a biological molecule of interest, a fluorescent marker, which is able to bind the target molecule, has to be introduced. The setups found in the revised works, which perform fluorescence imaging, also include a mono-chromatic light source, typically a UV LED, to generate the dye excitation and a smartphone camera used as detector to collect and measure the fluorescence intensity. The presented systems, proposed for a specific detection application, have the advantage of being applicable to other targets of interest through the use of different specific molecular dyes.

**Figure 2:**
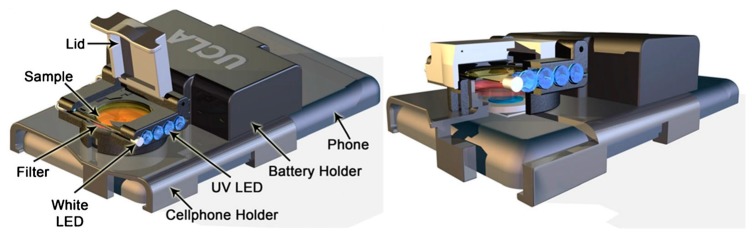
Schematic overview of the cellphone attachment for fluorescence diagnostics developed by Ludwig et al. [14]. Adapted with permission of Springer.

Zhu et al. [11] developed a portable *Escherichia coli* detection platform for screening of water and food samples. The cellphone-based fluorescence imaging platform was specifically realized to quantify the bacterial concentration in a water sample loaded into glass capillaries, opportunely functionalized with antibody directed against *E. coli*. Secondary antibodies, conjugated with quantum dots, were subsequently dispensed into the capillaries and served as the fluorescence signal. Quantum dots are inorganic nanocrystals with unique optical and chemical properties that give them exceptional brightness and photo-stability. In this case, UV-LEDs provided the excitation signal. The emission from the quantum dots through an additional lens was conveyed to the phone camera unit. A light-weight (~28 g) and compact (3.5 cm × 5.5 cm × 2.4 cm) attachment to the existing camera unit of a cellphone was designed to host the diagnostic platform. By quantifying the fluorescent light emission from each capillary tube, the concentration of *E. coli* in the sample was determined. The authors reported that the test can be completed within 2 h, including sample preparation, sample loading and incubation, with a detection limit of 5–10 CFU mL^−1^ in buffer solution. They also demonstrated the efficacy of this approach using fat-free milk as a matrix, where a similar detection limit was achieved. The same concept was used by Ludwig et al. [12] for the detection of the presence of anti-recombinant bovine somatotropin (rbST) antibodies in milk, which are endogenously produced upon administration of rbST, a milk production enhancer in dairy cattle which is illegal in the EU and represents a public health concern in the US. To monitor the presence of this biomarker, multiple immunoassay microspheres were used for the detection of multiple antibodies simultaneously within a small volume of a single sample. In addition to the same setup previously described, a white LED for dark-field imaging of all microspheres present in the sample was used. The cellphone-based diagnostic platform was successfully applied to milk sample extracts from rbST-treated and untreated cows. An 80% true-positive rate and 95% true-negative rate were achieved. The designed system can be adapted to any available cellphone that has a camera module simply by modifying the dimensions of the cellphone holder and 3D-printing another one accordingly (Figure 2). 

Mora et al. [13] developed a biosensor to accurately quantify lactose or galactose in undiluted food samples using genetically modified bacteria (*E. coli*) engineered to fluoresce in response to the analyte to reveal its diffusion behavior when using a blue-light source and optical filter. The authors reported detection limit concentrations in the range of 1–1000 mM requiring a sample volume of 1–10 µL and a storability of at least seven days at 4 °C without losing functionality. Bacteria possibily could be reprogrammed to serve as biosensors for other molecules. In this case, the smartphone is proposed as an alternative to visual inspection to quantify the fluorescent read-out.

#### 3.1.2. Smartphone-Based Colorimetric Readers 

Colorimetric assay is widely used in biochemistry to test for the presence of several analytes of interest, such as enzymes, antibodies, and peptides. It works by measuring the amount of light absorbed by a chromogenic reagent or a reaction product at a characteristic wavelength. This wavelength is specific to the reagent being measured. The amount of absorbed light is proportional to the concentration of reagent present in the assay well. Different solutions must be made, including a control solution for reference. In this section, the most used architecture is a smartphone camera used to detect the read-out from assay reactions.

**Figure 3:**
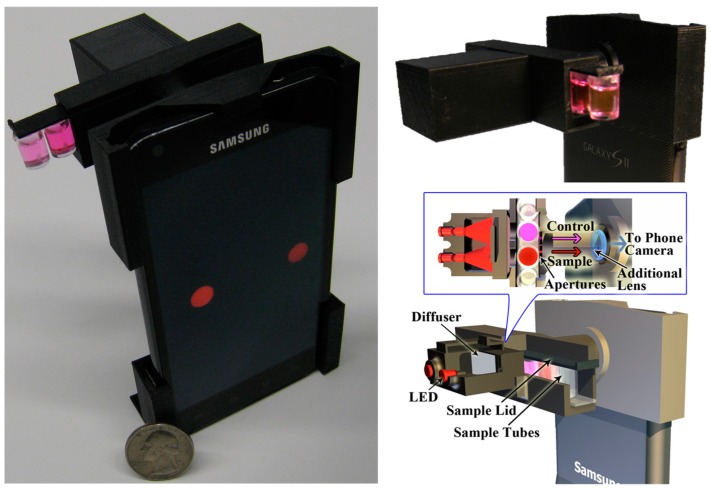
The iTube platform for performing cellphone-based colorimetric assays developed by Coskun et al. Adapted from [16] DOI: 10.1039/c2lc41152k with permission from The Royal Society of Chemistry. All rights reserved.

Coskun et al. [14] presented a cellphone-based system for colorimetric assays performed in tubes toward sensitive and specific detection of peanut allergen in food samples. They developed a cellphone attachment composed of two tubes, for the test and control solution, illuminated by two LEDs, whose wavelength matches the absorbance wavelength of the reagent activated in the test tube. The light intensity was measured by a dedicated application developed on the smartphone, to quantify the allergen concentration, after a calibration was performed with known concentrations of analyte within the test tube. The colorimetric assays were conducted based on an ELISA test kit specific to peanuts. A 20-min preparation phase was described for sampling and treatment of the target food sample to be ready for the digital reader implemented on the cellphone. The separate optical readout, optimized illumination and imaging configuration resulted to be sensitive, robust, repeatable and immune from manual reading errors compared to visual inspection which can be subject to variable light conditions. Weighing approximately 40 g, this digital tool was able to quantify peanut contamination in food samples with a minimum detection level of ~1 parts per million (ppm) (Figure 3). 

**Figure 4:**
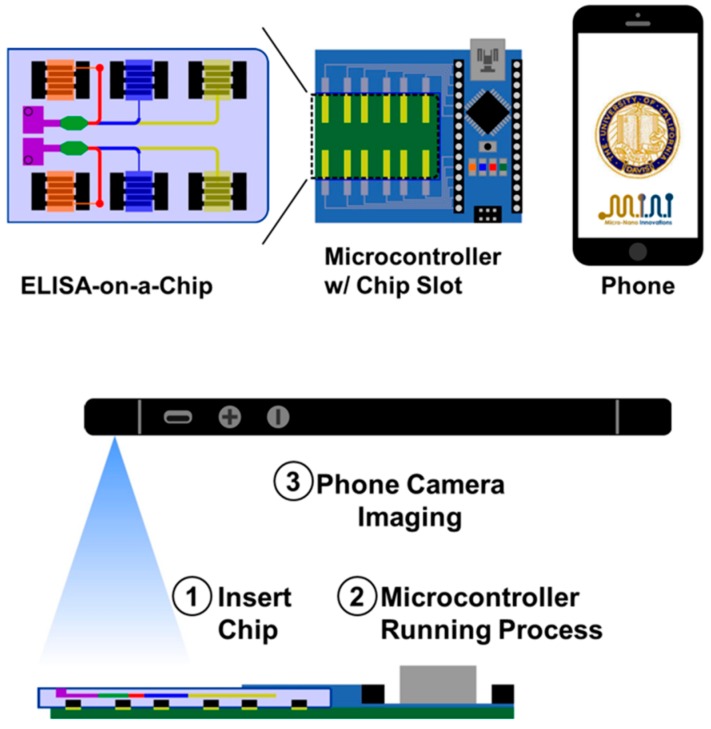
Schematic of the integrated mobile-interfaced diagnostic platform developed by Chen et al. Reprinted from [18], with the permission of AIP Publishing.

Lee et al. [15] developed a simple, rapid, and accurate smartphone-based lateral flow immunoassay (LFIA) reader for diagnosis of aflatoxin-B1 in maize. Aflatoxins are toxic secondary metabolites produced by a species of corn fungi. The Lateral Flow Immunoassay (LFIA) is a paper strip-based method for the detection and quantification of analytes. A liquid sample containing the analyte of interest moves under capillary action through various zones of strip, on which molecules that can interact with the analyte are attached. Starting from one end, the sample flows along the strip and it is bound by specific antibodies conjugated with colored or fluorescent particles, finally arriving at the other end of detection in which the recognition takes place, whose read-out is detectable by visual inspection or dedicated reader. In this work, a Samsung Galaxy S2 Smartphone is used as LFIA reader together with a close-up lens and a white LED, to improve the detection limit and sensitivity of the LFIA for AFB1 in maize, minimizing the read-out errors caused by visual inspection. The final result did not rely on the subjective interpretation of an operator. Combining microfluidics and competitive ELISA, Chen et al. [16] developed a smartphone-based portable system for the mobile detection of BDE-47, a common environmental contaminant in food samples. Through the USB port, the smartphone powered an Arduino Nano microcontroller integrated with a PCB, which in turn drove current to a microfluidic-based ELISA chip, triggering the analyte-mediated reaction. The colorimetric read-out was then acquired by the smartphone camera, and the image is then wirelessly transferred to a computing server for post-processing. This lab-on-chip assay showed a significant faster readout time of 15 min compared to conventional ELISAs which typically take at least 2 h. The input sample volume was considerably reduced with respect to laboratory ELISA. This allows the device to be field-deployable in a point-of-care to analyse less-than-ideal samples that the conventional method is insensitive and incapable of detecting (Figure 4). 

**Figure 5:**
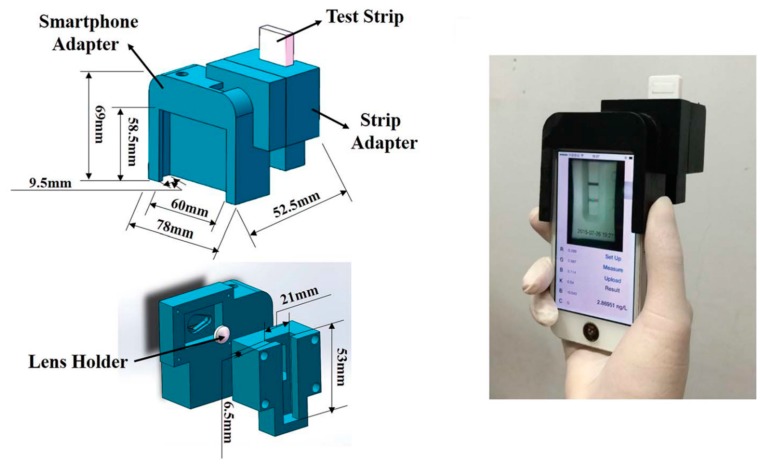
The on-site marine toxins diagnostic adapter developed by Fang et al. Adapted from [24] DOI: 10.1039/c2lc41152k with permission from The Royal Society of Chemistry. All rights reserved.

Park et al. [17] proposed a portable quality-control method for appraising red wine properties, by means of paper microfluidic channels and colorimetric assay performed with chemical dyes. Six different red wines were used as “model sample set” and four red wines were tested as “evaluation sample set”. A smartphone was used to acquire and analyse the colorimetric result, later processed by means of PCA analysis. Successful distinction of red wines by their grape varieties and oxidation was accomplished. PC1 was interpreted as explaining the sweetness (sweet or dry), while PC2 the body (light or heavy) of red wine. Minimization of sample-to-sample variation by splitting a single, undiluted red wine sample into eight different wells and filtering particulate matters by paper improved the reproducibility and led to smaller errors, resulting in better separation in the PCA plot. Such image processing and PCA can eventually be implemented as a stand-alone smartphone application, or within a cloud computing environment. Bueno et al. [18] developed a non-destructive method to discriminate three amines, as a diagnostic approach to detect foodborne pathogens in meat, combining membrane technology, dyes, chemometric tools and smartphone technology. A colorimetric test was evaluated using a smartphone and unsupervised chemometric tools, PCA and HCA, achieving a detection limit down to 1 ppm concentration of amine. To demonstrate the effectiveness of the proposed system in a real sample, sample meat were adulterated with amines and then analysed, but to infer the presence of bacteria, authors foresaw the necessity to test the biogenic amine production profile. With the aim of detecting antibiotic residues in milk, Masawat et al. [19] described the development of a smartphone-based digital image colorimeter. Although this work is not properly based on the use of biosensors, the analysis proposed here involved a sample pre-treatment procedure by using invasive Solid-Phase Extraction (SPE) technique to isolate and concentrate analyte of interest, tetracycline (TC), from the liquid matrix in which was dissolved. To protect the system from outside light, a photography lightbox was made with the internal walls sprayed with black paint. The TC solution filled in a quartz cuvette was located in a sample cell holder under fluorescent light inside the lightbox. An iPhone model was used for capturing digital images from outside the box via a drill hole, and the ColorConc application was used to analyze the images. A software calibration phase was performed with a set of images obtained from reference concentrations. Thus, the Euclidean distance algorithm was used to find the closest match to the given image, to identify sample concentration. Results were compared with double-beam UV–Vis Spectrophotometer. Obtained Limit of Detection (LOD) and Limit of Quantitation (LOQ) for TC concentration measurement, 0.50 and 1.50 µg mL^−1^, respectively, were higher than the Maximum Residue Limit (MRL) of TC in milk (0.1 µg mL^−1^). Thus, the pre-concentration of the sample in milk with SPE is necessary to detect TC at low concentration, with the effect of losing portability and ease of use of the proposed system. Monosik et al. [20] presented a paper-based colorimetric assay for the analysis of selected food compounds, instant soups and wines. Food samples were treated with a glutamate-specific enzyme, and colorimetric analyses were conducted after taking a picture with a smartphone, using freeware ImageJ. As a result, a limit of detection of 0.028 mmol L^−1^ was obtained, while for the naked eye the limit was 0.05 mmol L^−1^. The versatility of the proposed approach was demonstrated by using other enzymes from the same family. The described method did not require sophisticated approaches in terms of paper pre-treatment and very low volumes of sample and reagents are necessary for the analysis. Yu et al. [21] developed a portable sensing device for pathogen indirect detection based on a disposable lateral flow-through strip sensitive to alkaline phosphatase (ALP). ALP is an enzyme present in raw milk. It is slightly less labile to heat than most pathogenic bacteria; thus, loss of ALP activity is used to confirm proper pasteurization of skimmed or whole milk. The sample solution containing a desired concentration was added onto the sample pad. The read-out on the strip testing zone was imaged by a smartphone-integrated digital camera to quantify the optical signal. The images then were further analysed by a home-programmed MATLAB code. A trace amount of ALP as low as 0.1 U L^−1^ was distinguished within 10 min, with a detection range of 0.1–150 U L^−1^. Since the MATLAB code can be programmed to a mobile app, the analysis routine can be automated and performed on smartphone. Okadaic acid (OA) and saxitoxin (STX) are common marine toxins that can accumulate in shellfish and affect human health through the food chain. To avoid poisoning incidents, Fang et al. [22] proposed an on-site diagnostic platform using a smartphone with competitive immunoassay strips. A smartphone was employed for image acquisition and data processing. A 3D-printed portable accessory of the smartphone was used to fix the test strips. A homemade app was implemented for analysis. First, calibration curves were established with the target analytes diluted into five values of concentration. Then, real sample experiments were performed using the homemade strips for OA and STX and a commercial kit. By means of the strip adapter, the read-out was acquired using an optimized setup for light collection by the smartphone camera and analyzed. The proposed method showed a detection limit of 2.800 ng mL^−1^ for OA and 9.808 ng mL^−1^ for STX, similar to those of the commercial plate kit. The entire test time was 30 min, and the system was easy to operate, allowing on-site analysis with a low response time (Figure 5). 

**Figure 6:**
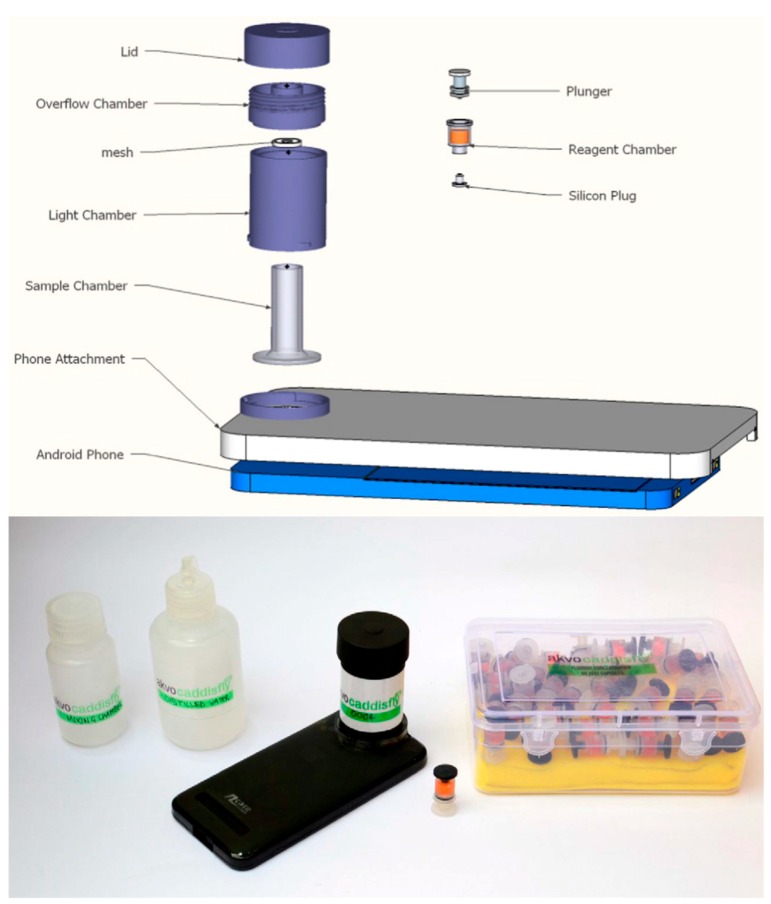
Schematic and picture of the smartphone-based fluoride test proposed by Levin et al. Adapted from [25], http://dx.doi.org/10.1016/j.scitotenv.2016.01.156 under the Creative Commons license http://creativecommons.org/licenses/by/4.0/.

Driven by the Indian issue of endemic fluorosis, a chronic disease resulting from excess intake of fluoride, Levin et al. [23] presented a field deployable colorimeter for screening of groundwater for fluoride in endemic areas. The proposed method used a commercially available reagent and adapted a smartphone as a colorimeter. An easy-fit, compact, sample chamber adapter for a smartphone was designed to optimize the colorimetric reading. The authors used three smartphones; calibration of each phone was necessary because of significant variation in colour sensing between different camera hardware. The calibration process involved analysing five different fluoride standards. A software program was developed to use with the phone for recording and analysing the RGB colour of the picture. The resulting images were analysed using a linear interpolation to calculate the expected colours in between the calibrated colours. The linear range for fluoride estimation was 0–2 mg L^−1^; results were comparable with those of expensive laboratory Ion Selective Electrode reference method, without the need for technical expertise to conduct the test analysis (Figure 6). 

Wang et al. [24] developed a smartphone-based colorimetric reader coupled with a remote server for rapid on-site analysis of catechols, an environmental pollutant highly toxic by ingestion and contact, which can irritate the human eyes and skin, and even at low concentrations, can give foods an undesirable taste. A 96-well sensor array was inserted in a light-tight box between a white LED light in the bottom and a smartphone fixed to the top. With this equipment, the ambient lighting condition and imaging distance/angle were kept constant when capturing the images of the sensor array. Thirteen different catechols at six serial concentrations were evaluated for system calibration using PCA, HCA and LDA for quality discrimination and PLS for quantitative determination. Data were uploaded to a remote server to form analysis polynomials for LDA and PLS of an unknown sample. Real water sample analysis was performed, with very good estimation results achieved. The authors claim this work to be the first dealing with the on-site detection of analytes using a smartphone-based colorimetric reader coupled to a remote server. With the aim of separating the detection means from the phone to resolve the difficulties in applying different models of mobile devices to the field test, Seo et al. [25] realized a pocket-sized immunosensor system for the on-site detection of foodborne pathogenic bacteria. The immunoassay procedure was based on chemiluminometric signal generation. The biosensor cartridge included a lens-free CMOS image sensor (CIS) physically contacting the signal generation part of the cartridge and Wi-Fi module installed in the circuit board. The system was controlled by a smartphone app programmed by the authors. The internet-of-things (IoT) technique was intended for use in food contamination monitoring and was demonstrated by analyzing *V. parahaemolyticus* present on fish samples and uploading the data to a server via a wireless network. Prior to food testing, the target bacterium was pre-cultivated. The cultured medium then was analysed by employing the immunosensor system controlled by the mobile device, and the result was uploaded as information to an internet server. A LoD of 1.4 × 10^4^ CFU mL^−1^ was achieved. Such a technique combining a biosensor with IoT can be used to issue a warning immediately after complete analysis about food contamination before purchase or consumption, so that the supply chain can be promptly blocked. The authors claim the study to be the first exemplification of pathogen monitoring via IoT. Finally, DuVall et al. [26] presented a rapid detection of foodborne pathogens using a cell phone and custom-written app, in which the physical identification was made by pathogen DNA transduction, mediated by magnetic bead aggregation with pathogenic DNA fragments. The smartphone was used to acquire picture of the assay reaction and analyse the image to perform a qualitative Yes or No detection of pathogen presence. The proposed detection modality was fully portable for point-of-care detection of food-borne pathogens *Escherichia coli O157:H7* and *Salmonella enterica*.

#### 3.1.3. Smartphone-Based Electro-Analytical Platforms

Electroanalytical methods use electrodes to make electrical contact with the analyte solution, in conjunction with electric or electronic devices to which they are attached, to measure an electrical parameter of the solution. The measured parameter is related to the quantity of an analyte in solution. According to the electric parameters that are measured, electroanalytical methods include potentiometry, amperometry, conductometry, electrogravimetry, voltammetry and coulometry. The names of the methods reflect the measured electric property or its units. Electroanalytical methods are particularly interesting for the development of smartphone-based platforms for on-site food diagnostics, as they combine high-performance detection with great simplicity, low-cost, portability, autonomy, cable-free operation, and capacity to conduct in real-time the entire analytical measurement at remote places.

**Figure 7:**
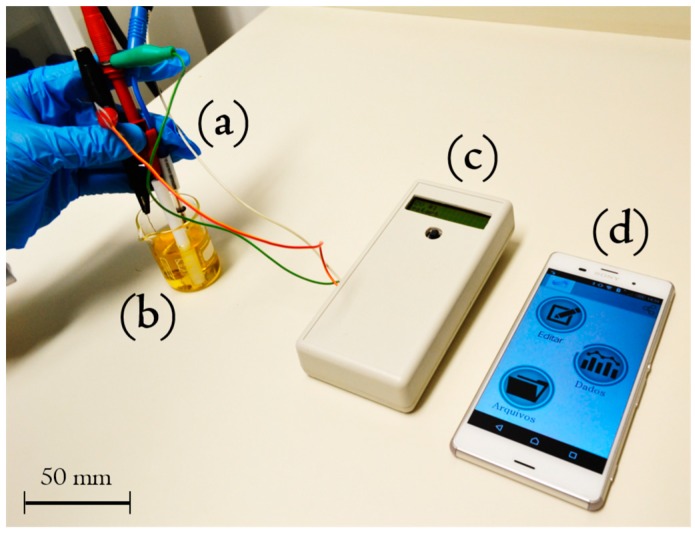
Portable platform deployed for point-of-use analyses. Electrochemical system. (**a**) Sample; (**b**) hand-held potentiostat; (**c**) and smartphone; (**d**) Reprinted from [29] with permission from Elsevier. http://www.sciencedirect.com/science/article/pii/S0013468616320400.

Dou et al. [27] described a biosensing system for the detection of clenbuterol (CLB), using a mobile electrochemical device with an electric field-driven acceleration strategy. CLB has been illegally used in livestock raising to improve growth rate, reduce fat deposition and increase protein accretion. However it has been banned as a feed additive in food-producing animals in most countries because it can easily remain in animal tissues and result in clinical symptom in human such as temporary dizziness and palpitations. The electric field-driven method was selected to accelerate the immunoreaction at the solid-liquid interface of electrodes, speeding up the transport of low-abundant drug molecules. A smartphone tool biochip was developed to conduct the electrochemical detection and send data to the phone via USB port. The smartphone-based immunosensor was able to detect a minimum of 0.076 ng mL^−1^ CLB in 6 min. The advantage of this method is that, by combining different functionalized electrodes, this device can meet the requirements for field detection of all food security-related species. In another study, Giordano et al. [28] coupled a homemade potentiostat to a mobile phone for point-of-use assays successfully applied for pattern recognition of Brazilian honey samples according to their botanical and geographic origins. The method relied on the unsupervised technique of principal component analysis (PCA), and the assays were performed by cyclic voltammetry using a working electrode of gold. The proposed biosensor platform was provided with both USB connection and Bluetooth module integrated in the potentiostat hardware. An in-house app was developed to ensure the on-site processing of multivariate data using PCA. The system was also created with the possibility to share data through the cloud (e-mail, Google drive, or even social media) for backup or remote processing of the electroanalytical results with more advanced chemometric tools. The authors claim that this is the first reported work concerning the development of a totally integrated point-of-use system with chemometric data processing on a smartphone (Figure 7).

### 3.2. Smartphone Spectroscopy

The works reviewed in this section belong to the optical diagnostics macro-category. Unlike the approach based on biosensors, in which it is necessary a reagent to trigger the transduction, in this case the analysis is performed in a non-invasive manner. In particular, spectroscopy has been a powerful tool in research and industrial applications. It is extensively and successfully used in applications including diagnostics, assessment of food quality, environmental sensing, and drug analysis testing. This technique is intrinsically rapid and non-destructive. However, most spectrometer setups used in industrial or laboratory-based applications are expensive and bulky, limiting them to controlled laboratory settings. Recently, due to advancements in electronics and fabrication methods, more portable spectrometers have been realized. Technological progress has allowed the release of micro-spectrometers which take advantage of new micro-technologies such as microelectromechanical systems (MEMS), micro-opto-electromechanical systems (MOEMS), micro-mirror arrays, etc. These improvements reduce cost and size while allowing good performance and high-volume manufacturability. Compared to lab-based instruments, miniaturized systems must become a black-box, providing expected results with high reliability and without intervention of technicians specialized in spectroscopy measurements. The ultimate goal, in the future, is the integration of a spectrometer into a smartphone, taking advantage of the highly efficient processing abilities in the compact configuration, to offer spectroscopic information on the fly. Moreover, they will use the large community of users to build databanks based on machine learning through apps [30]. A detailed overview of the revised Smartphone Spectroscopy works is provided with Table 2.

**Figure 8:**
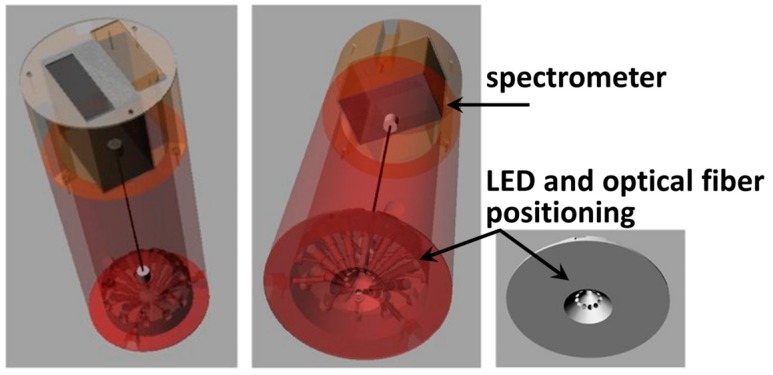
Rendering of the spectrometer-based colorimeter SpiderSpec. Reproducted from [31] with permission from SPIE.

Liang et al. [29] proposed a detection method for microbial spoilage of beef by means of a smartphone-based optical diagnostic system. An 880 nm near infrared (NIR) LED was irradiated perpendicular to the surface of ground beef, while the digital camera of a smartphone detected the scatter signal angled at 15°, 30°, 40°, and 60° from the incident light. Experiments were performed with and without positioning stagwhere in the latter case, a software application and the built-in gyro sensor of the smartphone were used to control the incidence angle between the iPhone camera and the NIR LED light source. Concentrations of *E. coli* (from 10^1^ CFU/mL to 10^8^ CFU/mL) were determined by the “pattern” of such scatter intensities over the angles. The proposed device was presented as a preliminary screening tool to monitor microbial contamination of meat products. Mignani et al. [31] presented the proof-of-concept of SpiderSpec, a compact colorimeter composed of a 3D printed cylindrical housing containing a LED array for illumination and a compact spectrometer for detection, with food control proposed as a possible application. The 12 visible LEDs were arranged in a circular array in the optical head, with 45° orientation with respect to the central detection axis, which is one of the standard configuration for reflectance measurements. The chosen spectrometer had an operative range of 350–800 nm. A custom Labview software interface was used for managing the LEDs and spectrometer. However, the authors, in a view of configuring the spectroscopic colorimeter as an IoT device, depicted future development of functionalities which could be selected using a smartphone or a tablet (Figure 8).

**Figure 9:**
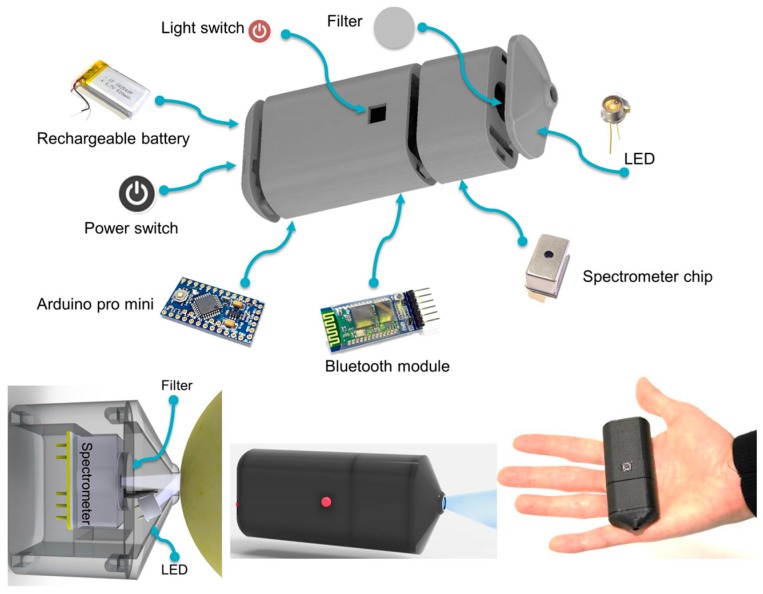
Schematic of the different components of the smartphone spectrometer prototype. Adapted from [35]. Published online 8 September 2016. doi:10.1038/srep32504, under the Creative Commons license http://creativecommons.org/licenses/by/4.0/.

Since conventional Fourier transform infrared (FTIR) spectrometers equipped with Attenuated Total Reflection (ATR) are bulky and expensive apparatus, on-site measurements of foods or drinks on the manufacturing site are impractical. In this perspective, Hosono et al. [40] developed an ultra-compact alkaline battery-size FTIR spectroscopic imager for simultaneous measurement of glucose and ethanol in alcoholic beverages by means of independent component analysis, employing a bean-size spectroscopic module to be mounted on smartphones. Experiments were performed in the NIR and MIR regions to find a range suitable for independent component analysis for discrimination of glucose and ethanol. The first use of a compact standalone spectrometer in combination with a smartphone via wireless connection was by Das et al. [32], which demonstrated the development of a mobile device for fruit ripeness evaluation. The authors used the portable spectrometer prototype to study UV fluorescence of chlorophyll (ChlF) in fruits. ChlF is a good indicator of photosynthetic activity and has been observed to relate to defects, damage, senescence and ripening of post-harvest fruits. Most important, this method enables the detection of fruit ripeness in a non-destructive manner. In this work, the smartphone spectrometer assembly was used to rapidly evaluate ripeness of different varieties of apples using ChlF emission when excited using UV light. UV LED with a wavelength of 360–380 nm was used as excitation source coupled with a spectrometer of range 340–780 nm. A calibration equation was applied to convert pixels to wavelength. Subsequently, a Bluetooth interface was setup to communicate with the smartphone. A customized app was developed for the Android operating system to communicate with the spectrometer assembly, and plot and analyse the spectra on the smartphone. ChlF detection in a variety of apple samples was performed and compared with the reference ripeness estimation using destructive mechanical firmness testing. The proposed device overcame the problem of stray light interference by launching and collecting light through a nozzle-like enclosure, thereby shielding any stray light contribution, a feature essential for field-based applications. However, a limiting factor in the proposed setup was the relatively low ADC bit resolution, property-dependent on the microcontroller choice (Figure 9).

**Figure 10:**
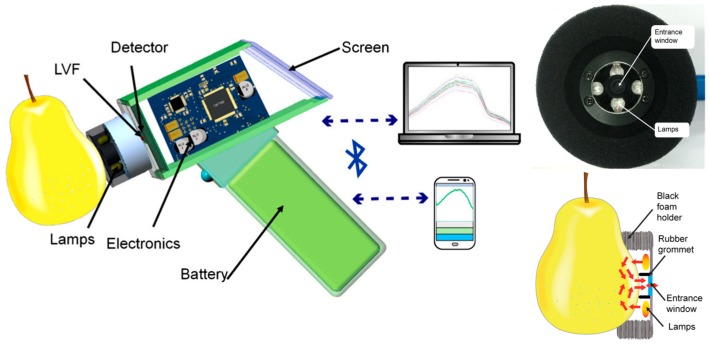
Schematic of the hand-held spectrometer and picture and schematics of the measuring head for interactance mode measurement developed by Yu et al. [39]. Adapted by permission of SAGE Publications, Ltd.

Contaminated foods originating from animal products are a significant source of human infection and illness. Because animal feces are the most likely source of pathogenic *E. coli* contamination associated with foodborne illnesses, it is particularly important to inspect for fecal contamination on meat during meat processing. Currently, meat inspection in slaughter plants for food safety and quality attributes, including potential fecal contamination, is conducted by visual examination from human inspectors. Oh et al. developed a handheld fluorescence-based imaging device to be an assistive tool for human inspectors with the aim of enhancing visual detection of fecal contamination on red meat, fat, and bone surfaces of beef under varying luminous intensities [33]. The device comprised four 405-nm 10 W LEDs for fluorescence excitation, a charge-coupled device (CCD) camera, an optical filter at 670 nm, and a Wi-Fi transmitter for sending real-time data to smartphone or tablet. The localization of most fecal contamination spots on beef surfaces was successfully identified because of the presence of chlorophyll metabolites discharging fluorescence near 670 nm. The image acquired from the device was transmitted by Wi-Fi and processed by MATLAB analysis. As expected, the increase in luminous intensities led to a parallel decrease in the identification of the fluorescence spots. Results indicated the proposed system as an effective way to aid visual inspection for fecal contamination detection. Rissanen et al. demonstrated a mobile phone-compatible hyper-spectral imager based on a tunable MEMS Fabry-Perot interferometer for authentication, counterfeit detection, and potential health/wellness and food sensing applications [34]. The authors described the development of a MEMS Fabry-Perot interferometer (FPI) tunable optical filters integrated with an iPhone 5s camera to perform hyper-spectral imaging in the vis-NIR range 450–550 nm. The communication between the MEMS FPI module and iPhone 5 was arranged using Bluetooth. A configuration of two cascaded FPIs (λ = 500 nm and λ = 650 nm) combined with an RGB colour camera showed potential to expand the wavelength tuning range to 400–700 nm. Sasikumar et al. [35] developed a handheld optical analyser consisting of a collimated 5 mW semiconductor red laser (635 nm) as the source, a circular spatial filter, Si detectors, and a Polydimethylsiloxane (PDMS) device. Fabrication of the PDMS device with integrated sample well was adapted for refractometric, and hence concentration, measurements. Potential integration of this device with smartphones was outlined, and featured a simple interface based on transmission mode configuration to explore several applications in food quality testing. Yu et al. realized a handheld NIR spectrometer specifically designed to assess the internal quality of fruit. In particular, a key development aspect was the Linear Variable Filter module as a light-dispersion component [36]. The proposed spectrometer system was a gun-shaped device, operating in the vis-NIR range (620–1080 nm) in interactance mode. The light source consisted of four tungsten lamps placed symmetrically around the entrance window. A 6-mm-diameter rubber grommet surrounded the entrance window and acted as a light seal, thus preventing surface-scattered light from reaching the window directly. A soft black foam ring around the detector head was provided to support the fruit during analysis and shield it from external light. The platform was tested for determining the sugar content in Crown Pear. Light from the source entered the fruit and penetrated part of the tissue, and that which emerged from the fruit entered the window. Results were compared with the reference method of Brix measurements, recorded with a handheld refractometer. Models were developed using PLS regression with the full band of the absorbance spectra and were optimised by applying MSC, SNV, and first derivative. The instrument was able to analyse spectral data using an on-board prediction model and to operate wirelessly with a smartphone, tablet or laptop computer. It proved highly suitable for predicting fruit internal quality. However, modified software is needed, and further studies are required to test the performance of the spectrometer for predicting other attributes or detecting sugar in other fruits (Figure 10).

To get an idea of the cost-reduction when switching to smartphone-based sensors, approximate costs are provide with Table 3, for some of the technologies above mentioned. 

## 4. Emerging Market of Smartphone-Based Food Diagnostic Platforms 

Many start-ups that are proposing the use of mobile devices able to test the quality of food and to determine its constituents are emerging, also thanks to the increasing use of the crowdfunding platforms Indiegogo and Kickstarter. These smart systems represent a mobile and miniaturized labs, optimized for the detection of a specific target, which are offered in combination with dedicated smartphone applications that provide friendly user interfaces for handling and displaying the test results, received through BLE (Bluetooth low energy) connection. Moreover, thanks to the ubiquitous smartphone connectivity, they become IoT modules able to leverage the extensive computational power and storage offered by cloud computing. Here, we provide the most relevant products, narrowing the list to those covered by a filed patent.

Cellmic LLC (formerly Holomic LLC), founded by Professor Aydogan Ozcan, offers a suite of rapid diagnostic test readers for advanced mobile diagnostics [37]. Among these is the allergen testing platform already described in this paper [16] and covered by a patent [38]. This device was developed by UCLA researchers for the detection of allergens, based on ELISA kit and a test tubes-containing module attachment to the smartphone camera. MyDx Inc. is a science and technology company that has created MyDx, a handheld electronic analyser that leverages electronic nose nanotechnology to accurately measure chemicals of interest in food and water, to detect traces of pesticides or metals, and send results to smartphone handled by the MyDx app [39]. The company owns many related patents including [41].

Scientists and researchers of Kaunas University of Technology, in cooperation with the company ARS Lab, have developed the patented [42] smart electronic nose FOODsniffer (formerly PERES). It is based on gas sensors and is intended to signal the deterioration of meat and fish by detecting gases that reflect such deterioration. It was selling on the company website for $129.99 at the time of writing [43]. Nimasensor [44], was developed by Nima Labs Inc. and enables the detection of the presence of gluten in food. It is based on the immunosensor technique, in which a specific antibody binds gluten, sparking the transduction process. Therefore, it is necessary to sample the food inside a disposable cartridge that is then inserted into the main body of the device. Results are provided through a dedicated app. The Nima device embeds an OLED display indicating a smile for gluten under 20 ppm. The Nima Starter Kit cost at the time of writing was $279.00 [45].

**Figure 11:**
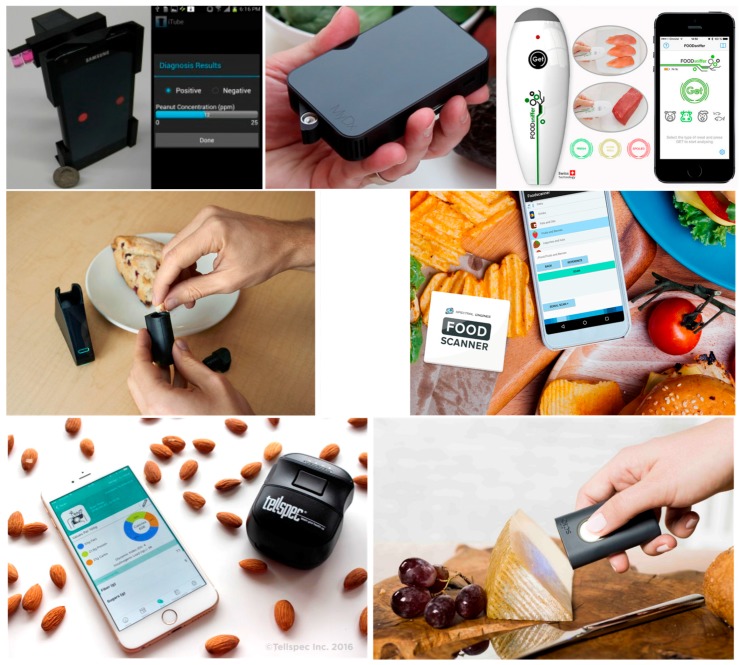
Commercial products for mobile food diagnostics.

Many new companies are offering sensors based on the emergent and promising technique of NIR spectroscopy. Spectral Engine Oy presented a plethora of high-tech products based on a tunable optical filter as a peculiar component, originated from years of research done at VTT Technical Research Centre of Finland, which resulted in many filed patents, including [46]. The Wireless NIR sensor device platform, which is designed for portable applications development, can be operated with a computer, a tablet, and a smartphone. The Food Scanner solution concept uses the wireless NIR sensor in conjunction with advanced algorithms, cloud-connectivity, and a vast material library to reveal the fat, protein, sugar, and total energy content of food items with a good level of accuracy [47]. TellSpec Inc. proposed a pocket-sized NIR spectrometer, a cloud-based patented [48] analysis engine, and a mobile app that work together to scan foods; identify calories, macronutrients, allergens, and contaminants; and provide relevant information such as food fraud, food adulteration, and food quality. The spectrometer is based on the Texas Instruments DLP^®^ NIRscan™ technology. At the time of writing, two solutions are currently delivered on the company website, the Enterprise Scanner at $1300.00 and the Software Development Kit at $2000.00 [49]. Finally, with several filed patents including the [50], the Israeli company Consumer Physics (formerly Verifood Ltd.), has the in-house-developed product, SCIO, a pocket-sized NIR spectrometer for molecular analysis, including food. It is delivered in the solutions of Consumer Edition at $299.00 and SDK at $499.00 at the time of writing. Moreover, in partnership with Changhong and Analog Devices, the company has just announced the world’s first Molecular Sensing Smartphone, a smartphone integrating the Scio spectrometer module [51]. The presented commercial devices are depicted in order of the description above in Figure 11.

## 5. Discussion

The proposed sensing strategies, which used a phone as the read-out tool (e.g., colorimetric and fluorescence imaging), are optimized for the phone models used for carried out the analysis. In order to meet the property of repeatability between different platforms, calibration of each phone is necessary because there is significant variation in color profile between different phones, due to hardware differences. Indeed, cameras may have different spectral responsivities, lamps may have different spectral emittances, and digitizer elements may be different. Moreover, the above mentioned properties may change over time. Another point to be addressed when dealing with pictures and colors is the image format. As smartphone cameras have become more and more powerful, it is now possible to shot in lossless RAW format, instead of lossy JPEG. In scientific imaging, there are several reasons to choose the first one. When shooting in JPEG image information is compressed and lost. The camera does its own processing to convert into a JPEG. The white balance and colour space are applied to the image by default. Drawback of this built-in processing is that it is clearly camera-dependent. With RAW, within the image all data from sensor are recorded, so it is always possible to perform post-processing, like adjusting white balance and selecting the proper colour space where to export out the picture. 

Sample preparation is still a bottleneck for the field of food mobile diagnostics, which aim is to bypass the use of expensive and bulky instrumentation-based tests, operated by trained personnel. Sampling performed by non-expert user may lead to unwanted contamination, resulting in defiled measurements. For example, in colorimetric assay, if unwanted solutes in the sample buffers positively or negatively affect light absorbance, it results respectively in false positives or negatives. This intrinsic drawback affects mostly detection strategies in which sampling is a necessary preliminary step. The commercial systems presented have tried to overcome this problem through a user-friendly design, inserting guidelines to assist customer during sampling and calibration procedure.

The methodologies discussed indirectly identify the concentration of a target substance, so for all of them it is necessary to make a calibration with a reference standard, using the pure substance at different concentrations, in order to build the instrument calibration curve for that specifically target. All conditions under which standards and unknowns are prepared should be kept identical. For fluorescence, dye- and glass-based reference materials are used to correct fluorescence emission for relative intensity, comparing an unknown measured intensity value with the certified values. In colorimetric assay, to correctly identify unknown samples, first the software module should be calibrated with a set of images obtained from reference concentrations. Each image will be associated with a concentration level. In case of electro-analytical techniques, the measured electrical quantity is proportional to the concentration of some component of the analyte. Calibration is done using standard buffer solutions at different known concentrations, developing the transduction characteristics. Reflectance spectroscopy needs calibration against a reference method with the ingredient of interest, to associate to a spectra a quantitative information. In the case of non-invasive analysis, such as reflectance spectroscopy, sampling is less important than performing a correct workflow for measurements. In the future, it is hoped that these systems will be able to self-calibrate. Non-invasive and machine learning-based systems, which rather than following standard calibration using reference concentrations, can count on huge amount of data, are definitely favorites from this point of view. Indeed, smartphone-based diagnostics allows ease capture of data and generation of large datasets, which can be appropriately managed by means of advanced computational analytics, such as machine/deep learning in combination with human expertise, for the extraction of meaningful information [52].

## 6. Conclusions

In this review, we have presented the recent developments in smartphone-based food diagnostics within the past 5 years. Results show that this novel field of research represents a promising area that has high scientific and commercial impact. In particular, advancements in biomedical science, chemistry, biotechnology, optics, and engineering have led to new diagnostic platforms which are more portable, economical and easier to use than conventional lab-based assays. Furthermore, the universal presence of mobile phones in our society makes it possible to leverage these devices for on-site testing. Nevertheless, these systems raise questions about use protocols and reliability of measurements. The repeatability of a measure, intrinsically guaranteed by a laboratory apparatus, becomes a delicate condition to be met in case of portable modules for on-site analysis. Opportune optimizations must be evaluated at the design stage for the physicality of the instrument, to exclude or minimize any external noise sources.

Researchers have come up with different solutions and embodiments for exploiting the great potential offered by smartphones. According to the detection strategy, we have classified the revised works into two main classes; biosensor-, and spectroscopy-based smartphone platforms. In both of these approaches, mobile phone provided a simplified user interface, visual display, data processing, storage, and wireless transmission. While both strategies feature advantages, they also face several limitations. Biosensors are economic and intrinsically sensitive to a specific target and only need slight computation processes. Nevertheless, they are based on disposable cartridges or strips, and require invasive sampling to perform the diagnostics. Spectroscopy indeed allows peculiar rapid non-invasive and non-destructive analysis. However, it is not a target-specific method and it must be associated with complex multivariate statistical and chemometric tools for spectral dataset analysis to extract the relevant chemical information. While in the biosensors based-approach, the key-enabling factors are the choices of the reagent and the transduction process, in case of spectroscopy, the critical design criteria are represented by the selections of the wavelength range for source and detector, and of the measurement setup. Thus, both approaches are easily adaptable methods, since calibration and tuning of the systems are performed according to the desired application. Moreover, they require only a basic training, and a few minutes for detecting and processing, with the potential for providing a user-friendly, on-the-go, scanning scenario. While lab-on-smartphone biosensor applications are well established, the exploitation of smartphone spectroscopy is in its infancy. Fortuitously, advancements in the fabrication of optical sensors, which are leading to increasingly miniaturized and economical technology, are keeping pace with the development of increasingly sophisticated machine-learning algorithms. This, combined with the enormous potential offered by cloud computing, and the ability of modern smartphones to act as both connecting portals and interfaces for analysis and display of results, dramatic developments are foreseen in the field of mobile diagnostics, operated not only for food monitoring applications, but also for environmental and biomedical sensing.

## Figures and Tables

**Table 1:** Summary of recent lab-on-smartphone biosensor platforms.

Detection Target	Methodology	Materials	LoD/Test Time/Performance	Smartphone Use	Reference
*Escherichia coli* in water	Fluorescent imaging	Antibody, quantum dots, UV LED	5–10 CFU mL^−1^	Cellphone imaging with camera attachment	[11]
rbST antibodies in milk	Microsphere fluorescent immunoassay	Antibody, quantum dots, UV LED and white LED	80% true-positive rate and 95% true-negative rate	Cellphone imaging with camera attachment	[12]
Lactose and galactose in undiluted food samples	Engineered bacteria fluorescence	Blue light and optical filter	1–1000 mM	Cellphone imaging	[13]
Peanut allergen in food samples	Colorimetric assays	ELISA allergen test kit, cellphone attachment with 2 test tubes and 2 LEDs	~1 parts per million (ppm), 20-min preparation phase	Cellphone assay with camera attachment	[14]
Aflatoxin B1 in maize	Lateral flow immunoassay	Paper strip, close-up lens and a white LED	5 μg/kg	Smartphone imaging via LFIA reader adapter	[15]
BDE-47 in food sample	Microfluidics and competitive ELISA	Arduino Nano, PCB, microfluidic chip	Readout time of 15 min and input sample volume considerably reduced	Smartphone as power source, imaging reader and cloud sender	[16]
Red wine properties	Paper microfluidics, colorimetric assay, and PCA	Chemical dyes	Successful distinction of red wines by their grape varieties and oxidation.	Smartphone imaging	[17]
Amines as indication of foodborne pathogens in meat	Membrane technology colorimetry and unsupervised chemometric tools	Dyes	Down to 1 ppm concentration of amine	Camera imaging	[18]
Antibiotic residues in milk	SPE and fluorescence spectroscopy	Photography lightbox with fluorescent light	LoC 0.50 mL^−1^ and LoQ 1.50 µg mL^−1^	Smartphone camera used as spectrometer	[19]
Glutamate in food compound, instant soup and wines	Paper-based colorimetric assay	Glutamate-specific enzyme	0.028 mmol L^−1^	Camera acquisition and analysis	[20]
ALP as indicator of incorrect milk pasteurization	Disposable lateral flow-through strip	Sample pad	0.1 U L^−1^, within 10 min with a detection range of 0.1–150 U L^−1^	Image acquisition and Matlab analysis	[21]
OA and STX in shellfish	Competitive immunoassay strip	3D-printed smartphone strip adapter	2.800 ng mL^−1^ for OA and 9.808 ng mL^−1^ for STX in 30 min	Camera acquisition via strip adapter and data processing	[22]
Fluoride in water	Colorimetric imaging	Compact sample chamber adapter for smartphone	Linear range 0–2 mg L^−1^	Smartphone colorimeter	[23]
Catechols in water	Colorimetric imaging	96-well sensor array, light-tight box, white LED	PCA, HCA and LDA for quality discrimination and PLS for quantitative determination	Smartphone colorimeter coupled to remote server	[24]
*V. parahaemolyticus* in fish samples	Colorimetric immunoassay	Biosensor cartridge, lens-free CMOS image sensor, Wi-Fi module	1.4 × 10^4^ CFU mL^−1^	Dedicated app to operate the system and upload on internet server	[25]
*Escherichia coli O157:H7* and *Salmonella enterica*	DNA transduction on microfluidic device	Magnetic beads	Down to 20 genomic copies of *E. coli*	Custom written app for cell phone image analysis	[26]
Clenbuterol	Electric field-driven immunoreaction	Functionalized electrodes	0.076 ng mL^−1^ CLB in 6 min	USB Smartphone tool biochip	[27]
Pattern recognition of Brazilian honey samples	Cyclic voltammetry assay	Electrode of gold, homemade potentiostat with USB connection and Bluetooth module	Successfully generation of voltammetric fingerprints of numerous honey samples	Chemometric data processing on smartphone	[28]

**Table 2:** Summary of recent smartphone spectroscopy systems.

Detection Target	Methodology	Materials	LoD/Performance	Smartphone Use	Reference
Microbial spoilage on beef	Mie scattering	Positioning stages, 880 nm NIR LED	10^1^ CFU mL^−1^ to 10^8^ CFU mL^−1^	Built-in gyro sensor and camera spectroscopy	[32]
Generic application	Spectroscopic colorimetry	3D printed housing, LED array, Phidgets board, and VIS-spectrometer	Good agreement to certified spectra with dE/E ranging from 0.5% to 1.5%	IoT device to be used with smartphone	[33]
Glucose and ethanol in alcoholic beverages	FTIR spectroscopy and independent component analysis	Graphite light source, ATR prisms, 2-dimensional light receiving device for smartphone	Wavelength resolution 0.057 μm	Proposed as a bean-size spectroscopic module to be mounted on smartphones	[34]
ChlF detection in a variety of apple samples	UV fluorescence spectroscopy	UV LED, nozzle-like enclosure VIS-spectrometer, Arduino pro mini µ, Bluetooth	Satisfactory agreement observed between ripeness and fluorescence signals	Dedicated app interface on smartphone to communicate, receive, plot, and analyse spectral data	[35]
*E. coli* contamination on meat	Fluorescence-based imaging	4405-nm 10 W LEDs, CCD camera, optical filter at 670 nm, and Wi-Fi transmitter	Localization of most fecal contamination spots successfully identified	Outlined real-time broadcasting to monitoring device such as smartphone	[36]
Generic food sensing application	Hyper-spectral imaging	Tunable MEMS FPI, Bluetooth	Operation range 450–550 nm with spectral resolution 8–15 nm @FWHM	Mobile phone-compatible hyper-spectral imager	[37]
Food quality testing	Diffractive interference refractometry	5 mW semiconductor red laser, circular spatial filter, Si detectors, and a PDMS device	LoD of 4 × 10^−4^ RIU	Outlined smartphone interface based on transmission mode configuration	[38]
Sugar content prediction in pears	NIR spectrometry and PLS	4 tungsten lamps, LVF 620–1080 nm and CMOS linear detector array	Low power, SNR ratio up to 5000, R^2^ 0.96, SEC 0.29° Bx and SEP 0.46° Bx	Instrument wirelessly operated with smartphone	[39]

**Table 3:** Approximate cost related to works which have provided an estimate.

Platform	Approximate Cost	Reference
Smartphone with fluorescence microscope attachment	Attachment of around $140, significantly reduced compared to the equipment costs for the reference method	[12]
Akvo Caddisfly	Expected to retail at $75, without the phone and mapping system, plus $0.3 for each test	[23]
PiBA assay coupled to LAMP	Reagent cost for PiBA is a fraction of a cent. Overall cost reduction is ~10-fold respect to the reference (fluorescence reagents for qPCR)	[26]
Smartphone-based analytical platform with homemade potentiostat	Based on CheapStat potentiostat which requires less than eighty dollars for its manufacturing, while the most commercial potentiostats cost a few thousands of dollars	[28]
Smartphone spectrometer	Entire assembly along with the smartphone can be realized under $250, while reference spectrometer platforms costs are $4000 and $1200	[32]

## References

[1] Horizon Prize Food Scanner 1. http://ec.europa.eu/research/horizonprize/index.cfm?prize=food-scanner.

[2] Ozcan A. (2014). Mobile phones democratize and cultivate next-generation imaging, diagnostics and measurement tools. Lab Chip.

[3] Wu M.Y.-C., Hsu M.-Y., Chen S.-J., Hwang D.-K., Yen T.-H., Cheng C.-M. (2017). Point-of-Care Detection Devices for Food Safety Monitoring: Proactive Disease Prevention. Trends Biotechnol..

[4] Zhang D., Liu Q. (2016). Biosensors and bioelectronics on smartphone for portable biochemical detection. Biosens. Bioelectron..

[5] Li F., Bao Y., Wang D., Wang W., Niu L. (2016). Smartphones for sensing. Sci. Bull..

[6] Daponte P., Vito L., de Picariello F., Riccio M. (2013). State of the art and future developments of measurement applications on smartphones. Measurement.

[7] Xu X., Akay A., Wei H., Wang S., Pingguan-Murphy B., Erlandsson B.E., Li X., Lee W., Hu J., Wang L. (2015). Advances in Smartphone-Based Point-of-Care Diagnostics. Proc. IEEE.

[8] Roda A., Michelini E., Zangheri M., Di Fusco M., Calabria D., Simoni P. (2016). Smartphone-based biosensors: A critical review and perspectives. TrAC Trends.

[9] Liu X., Lin T.-Y., Lillehoj P.B. (2014). Smartphones for Cell and Biomolecular Detection. Ann. Biomed. Eng..

[10] Yang K., Peretz-Soroka H., Liu Y., Lin F. (2016). Novel developments in mobile sensing based on the integration of microfluidic devices and smartphones. Lab Chip.

[11] Zhu H., Sikora U., Ozcan A. (2012). Quantum dot enabled detection of Escherichia coli using a cell-phone. Analyst.

[12] Ludwig S.K.J., Zhu H., Phillips S., Shiledar A., Feng S., Tseng D., van Ginkel L.A., Nielen M.W.F., Ozcan A. (2014). Cellphone-based detection platform for rbST biomarker analysis in milk extracts using a microsphere fluorescence immunoassay. Anal. Bioanal. Chem..

[13] Mora C.A., Herzog A.F., Raso R.A., Stark W.J. (2015). Programmable living material containing reporter micro-organisms permits quantitative detection of oligosaccharides. Biomaterials.

[14] Coskun A.F., Wong J., Khodadadi D., Nagi R., Tey A., Ozcan A. (2013). A personalized food allergen testing platform on a cellphone. Lab Chip.

[15] Lee S., Kim G., Moon J. (2013). Performance improvement of the one-dot lateral flow immunoassay for aflatoxin B1 by using a smartphone-based reading system. Sensors.

[16] Chen A., Wang R., Bever C.R.S., Xing S., Hammock B.D., Pan T. (2014). Smartphone-interfaced lab-on-a-chip devices for field-deployable enzyme-linked immunosorbent assay. Biomicrofluidics.

[17] Park T.S., Baynes C., Cho S.-I., Yoon J.-Y. (2014). Paper microfluidics for red wine tasting. RSC Adv..

[18] Bueno L., Meloni G.N., Reddy S.M., Paixao T.R.L.C. (2015). Use of plastic-based analytical device, smartphone and chemometric tools to discriminate amines. RSC Adv..

[19] Masawat P., Harfield A., Namwong A. (2015). An iPhone-based digital image colorimeter for detecting tetracycline in milk. Food Chem..

[20] Monosik R., dos Santos V.B., Angnes L. (2015). A simple paper-strip colorimetric method utilizing dehydrogenase enzymes for analysis of food components. Anal. Methods.

[21] Yu L., Shi Z., Fang C., Zhang Y., Liu Y., Li C. (2015). Disposable lateral flow-through strip for smartphone-camera to quantitatively detect alkaline phosphatase activity in milk. Biosens. Bioelectron..

[22] Fang J., Qiu X., Wan Z., Zou Q., Su K., Hu N., Wang P. (2016). A sensing smartphone and its portable accessory for on-site rapid biochemical detection of marine toxins. Anal. Methods.

[23] Levin S., Krishnan S., Rajkumar S., Halery N., Balkunde P. (2016). Monitoring of fluoride in water samples using a smartphone. Sci. Total Environ..

[24] Wang Y., Li Y., Bao X., Han J., Xia J., Tian X., Ni L. (2016). A smartphone-based colorimetric reader coupled with a remote server for rapid on-site catechols analysis. Talanta.

[25] Seo S.M., Kim S.W., Jeon J.W., Kim J.H., Kim H.S., Cho J.H., Lee W.H., Paek S.H. (2016). Food contamination monitoring via internet of things, exemplified by using pocket-sized immunosensor as terminal unit. Sens. Actuators B Chem..

[26] DuVall J.A., Borba J.C., Shafagati N., Luzader D., Shukla N., Li J., Kehn-Hall K., Kendall M.M., Feldman S.H., Landers J.P. (2015). Optical Imaging of Paramagnetic Bead-DNA Aggregation Inhibition Allows for Low Copy Number Detection of Infectious Pathogens. PLoS ONE.

[27] Dou Y., Jiang Z., Deng W., Su J., Chen S., Song H., Aldalbahi A., Zuo X., Song S., Shi J., Fan C. (2016). Portable detection of clenbuterol using a smartphone-based electrochemical biosensor with electric field-driven acceleration. J. Electroanal. Chem..

[28] Giordano G.F., Vicentini M.B.R., Murer R.C., Augusto F., Ferrão M.F., Helfer G.A., da Costa A.B., Gobbi A.L., Hantao L.W., Lima R.S. (2016). Point-of-use electroanalytical platform based on homemade potentiostat and smartphone for multivariate data processing. Electrochim. Acta.

[29] Liang P.-S., Park T.S., Yoon J.-Y. (2014). Rapid and reagentless detection of microbial contamination within meat utilizing a smartphone-based biosensor. Sci. Rep..

[30] Bouyé C., Kolb H., D’Humières B. (2016). Mini and micro spectrometers pave the way to on-field advanced analytics. Proceedings of the SPIE-Photonics Instrumentation Engineering III.

[31] Mignani A.G., Mencaglia A.A., Baldi M., Ciaccheri L., Lee B., Lee S.B., Rao Y. (2015). SpiderSpec: A low-cost compact colorimeter with IoT functionality. Proceedings of the SPIE Fifth Asia-Pacific Optical Sensors Conference.

[32] Das A.J., Wahi A., Kothari I., Raskar R. (2016). Ultra-portable, wireless smartphone spectrometer for rapid, non-destructive testing of fruit ripeness. Sci. Rep..

[33] Oh M., Lee H., Cho H., Moon S.-H., Kim E.-K., Kim M.S., Kim M.S., Chao K., Chin B.A. (2016). Detection of fecal contamination on beef meat surfaces using handheld fluorescence imaging device (HFID). Proceedings of the SPIE Sensing for Agriculture and Food Quality and Safety VIII.

[34] Rissanen A., Saari H., Rainio K., Stuns I., Viherkanto K., Holmlund C., Nakki I., Ojanen H., Druy M.A., Crocombe R.A. (2016). MEMS FPI-based smartphone hyperspectral imager. Proceedings of the SPIE Next-Generation Spectroscopic Technologies IX.

[35] Sasikumar H., Prasad V., Pal P., Varma M.M., Cote G.L. (2016). Diffractive Interference Optical Analyzer (DiOPTER). Proceedings of the SPIE Optical Diagnostics and Sensing XVI: Toward Point-of-Care Diagnostics.

[36] Yu X., Lu Q., Gao H., Ding H. (2016). Development of a handheld spectrometer based on a linear variable filter and a complementary metal-oxide-semiconductor detector for measuring the internal quality of fruit. J. Near Infrared Spectrosc..

[37] CELLMIC | We Innovate Mobile Diagnostics |. http://www.cellmic.com/.

[38] Ozcan A., Coskun A., Wong J. (2013). Allergen Testing Platform for Use with Mobile Electronic Devices. U.S. Pat. App..

[39] OrganaDx Sensor | Portable Food Tester Analyzer. http://www.cdxlife.com/organa-sensor/.

[40] Hosono S., Qi W., Sato S., Suzuki Y., Fujiwara M., Hiramatsu H., Suzuki S., Abeygunawardhana P.K.W., Wada K., Nishiyama A. (2015). Proposal of AAA-battery-size one-shot ATR Fourier spectroscopic imager for on-site analysis-Simultaneous measurement of multi-components with high accuracy. Proceedings of the SPIE 9314, Optics and Biophotonics in Low-Resource Settings.

[41] Rouse R. (2014). Apparatus for Detection and Delivery of Volatilized Compounds and RELATED Methods. U.S. Patent Application.

[42] Gailius D. (2014). Electronic Nose for Determination of Meat Freshness. U.S. Patent Application.

[43] FOODsniffer. http://www.myfoodsniffer.com/.

[44] Sundvor S., Portela S., Ward J., Walton J. (2016). System and Method for Detection of Target Substances. U.S. Patent Application.

[45] Nima-A Portable Gluten Tester | Nima. https://nimasensor.com/.

[46] Antila J., Kantojärvi U., Mäkynen J. (2016). Optical Measurement System. WO2016071572A1.

[47] Food Scanner. https://www.spectralengines.com/products/food-scanner.

[48] Watson W., Correa I. (2015). Analyzing and Correlating Spectra, Identifying Samples and Their Ingredients, and Displaying Related Personalized Information. US Patent.

[49] Tellspec–Beam Your Health Up–TellSpec. http://tellspec.com/en/.

[50] Goldring D., Sharon D. (2016). Low-Cost Spectrometry System for End-User Food Analysis. US Patent.

[51] Consumer Physics. https://www.consumerphysics.com/myscio/.

[52] Contreras-Naranjo J., Wei Q. (2016). Mobile phone-based microscopy, sensing, and diagnostics. IEEE J. Sel..

